# Denatonium Benzoate, the Most Bitter Compound, Reduces Weight by Promoting Adipocyte Browning

**DOI:** 10.3390/metabo16040242

**Published:** 2026-04-02

**Authors:** Yiqin Niu, Junhui Shao, Yanping Teng, Ce Zhang, Xin Xie, Shimeng Guo

**Affiliations:** 1 School of Pharmacy, Binzhou Medical University, Yantai 264003, China; 2 Shandong Laboratory of Yantai Drug Discovery, Bohai Rim Advanced Research Institute for Drug Discovery, Yantai 264117, China; 3 State Key Laboratory of Drug Research, National Center for Drug Screening, Shanghai Institute of Materia Medica, Chinese Academy of Sciences, Shanghai 201203, China

**Keywords:** denatonium benzoate (DB), obesity, white adipose tissue browning, Ucp1

## Abstract

Objectives: Obesity remains a global health challenge, and promoting white adipose tissue browning has emerged as a promising anti-obesity strategy. This study aimed to investigate the anti-obesity effects of denatonium benzoate (DB) and elucidate its underlying mechanisms. Methods: In order to study the anti-obesity effects of DB and its mechanisms, we used in vivo and in vitro obesity models to study whether DB has anti-obesity effects by participating in fat browning. We investigated the role of DB in high-fat diet (HFD)-induced obese *C57BL/6J* mice using 36 male animals (8 weeks old, 25 ± 2 g), and evaluated the expression of the adipogenic marker genes Fatty acid-binding protein 4 (*Fabp4*) and Peroxisome Proliferator-Activated Receptor gamma (*PPAR-γ*); the thermogenic genes uncoupling protein 1 (*Ucp1*), Transcription Factor A, Mitochondrial (*TFAM*), Peroxisome Proliferator-Activated Receptor Gamma Coactivator 1-Alpha (*Pgc1α*), and Adrenergic receptor beta 3 (*Adrb3*); as well as the adipose browning marker genes Deiodinase, Iodothyronine, Type II (*Dio2*), PR domain containing 16 (*PRDM16*), and Peroxisome Proliferator-Activated Receptor alpha (*PPAR-α*) in 3T3-L1 cells and primary adipocytes with DB treatment. Conclusions: These results indicate that the anti-obesity effects of DB may be related to the browning of white fat, providing a novel potential candidate for anti-obesity drug development.

## 1. Introduction

Obesity has emerged as a global health priority due to its escalating prevalence and strong association with a spectrum of serious complications, including type 2 diabetes and cardiovascular diseases [1]. According to the WHO 2024 [2] Global Status Report on Noncommunicable Diseases, the global prevalence of obesity in adults (18 years and older) reached 16% in 2022, with 2.5 billion adults overweight (43% of the global adult population); childhood obesity has increased dramatically, with 35 million children under 5 years old overweight or obese worldwide in 2024. This condition fundamentally arises from an imbalance between caloric intake and energy expenditure, thereby driving the excessive expansion of adipose tissue [3].

Adipose tissue comprises three distinct types: white adipose tissue (WAT), brown adipose tissue (BAT), and beige adipose tissue [4]. The primary function of brown adipocytes is thermogenesis, facilitated by the mitochondria-rich content of the uncoupling protein 1 (Ucp1). Ucp1 uncouples oxidative phosphorylation, converting energy into heat rather than synthesizing Adenosine Triphosphate (ATP) [5], making it the pivotal regulator of heat production [6]. Notably, mice deficient in BAT exhibit severe obesity [7]. In contrast, white adipocytes primarily serve as energy storage depots, with fewer mitochondria and consequently lower metabolic activity compared to brown adipocytes. However, under cold exposure or other stimuli, Ucp1-expressing mitochondria-rich adipocytes emerge within WAT [6]. These cells are termed “beige” or “brite” (brown with white) adipocytes. Beige fat is defined as Ucp1-expressing adipocytes that arise outside classical BAT depots. Similarly to brown adipocytes, beige adipocytes possess thermogenic capacity [6,8,9,10]. Previous studies have shown that BAT exerts beneficial metabolic effects in humans, with its quantity positively correlated with energy expenditure [11,12]. Therefore, inducing the browning of white adipocytes has emerged as a new anti-obesity target.

Denatonium benzoate (DB) is a strong bittering agent commonly added into household products to prevent the ingestion of harmful substances through taste aversion [13,14]. Notably, intragastric administration of DB has been reported to reduce body weight in healthy volunteers, suggesting its potential anti-obesity effects [15]. Other studies indicate that DB can reduce fat accumulation in differentiated adipocytes by increasing metabolic activity [16]. Beyond its role in weight management, emerging evidence suggests that DB exerts beneficial effects on lipid metabolism and oxidative stress. Preclinical studies have demonstrated that denatonium acetate (DA), a structurally related compound, significantly reduces circulating low-density lipoprotein (LDL) cholesterol, total cholesterol (TC), and triglycerides (TGs) in high-fat-diet-fed mice in a dose-dependent manner [17]. In a Phase 2 clinical trial, oral administration of ARD-101 (a gut-restricted formulation of DA) for 28 days resulted in reductions in LDL cholesterol (mean change: −6.8 mg/dL), total cholesterol (−4.4 mg/dL), and non-HDL cholesterol (−4.6 mg/dL) in adults with obesity, with particularly pronounced effects observed in participants with elevated baseline LDL levels (>130 mg/dL) [17]. Mechanistically, these lipid-lowering effects are mediated through the activation of bitter taste receptors (TAS2Rs), particularly TAS2R38, which stimulate the secretion of glucagon-like peptide-1 (GLP-1) from intestinal enteroendocrine L-cells [18]. GLP-1 subsequently enhances insulin sensitivity, suppresses hepatic gluconeogenesis, and promotes lipid clearance [19]. Furthermore, TAS2R activation by bitter compounds has been shown to upregulate the nuclear factor erythroid 2-related factor 2 (Nrf2) pathway, thereby enhancing cellular antioxidant capacity and reducing oxidative stress [20,21]. Specifically, TAS2R agonists promote Nrf2 nuclear translocation, leading to increased expression of antioxidant enzymes such as heme oxygenase-1 (HO-1) and superoxide dismutase (SOD), which mitigate reactive oxygen species (ROS) accumulation and protect against vascular inflammation [20,21]. Additionally, DB and related bitter compounds may modulate gut microbiota composition, increasing short-chain fatty acid (SCFA) production, which further contributes to improved metabolic profiles and reduced systemic inflammation [22,23].

However, it remains unclear whether DB plays a role in the differentiation of beige adipocytes. To investigate this, we tested the effect of DB in 3T3-L1 cells and primary adipocytes. We also explored whether long-term intragastric administration of DB influences the body weight of obese mice induced by a high-fat diet (HFD).

## 2. Materials and Methods

### 2.1. Reagents and Materials

Denatonium benzoate (DB, chemical formula: C_28_H_34_N_2_O_3_, Cat. No. T1098, purity ≥ 98%), insulin (Cat. No. T8221), dexamethasone (Cat. No. T6465), and 3-isobutyl-1-methylxanthine (Cat. No. T1713) were purchased from Tsbiochen Co., Ltd., (Shanghai, China). DMEM medium, fetal bovine serum (FBS), and penicillin-streptomycin (P/S) were purchased from Gibco Co., Ltd., (Grand Island, NY, USA). Collagenase enzyme was purchased from Sigma-Aldrich Co., Ltd., (St. Louis, MO, USA). Oil Red O staining kit was purchased from Beyotime Biotechnology Co., Ltd., (Shanghai, China). RIPA lysis buffer, protease and phosphatase inhibitors, and Bradford protein assay kit were purchased from Thermo Fisher Scientific Co., Ltd., (Waltham, MA, USA). Antibodies against Ucp1 (Cat. No. ab10983), *PRDM16* (Cat. No. ab106410), and GAPDH (Cat. No. ab181602) were purchased from Abcam Co., Ltd., (Cambridge, UK). TRIzol Reagent (Cat. No. R401-01), 5× All-in-One RT Master Mix (Cat. No. R333-01), and 2× ChamQ Blue Universal SYBR qPCR Master Mix (Cat. No. Q312-02) were purchased from Vazyme Biotech Co., Ltd., (Nanjing, China). Mouse GLP-1/CCK ELISA kits were purchased from Enzyme-linked Biotechnology Co., Ltd., (Shanghai, China). High-fat diet (Research Diets, Cat. No. D12492) was purchased from Shanghai Bioleaf Biotech Co., Ltd. (Shanghai, China). All other chemical reagents were of analytical grade and purchased from Sinopharm Chemical Reagent Co., Ltd., (Shanghai, China).

### 2.2. Experimental Animal

Male *C57BL/6J* mice (8 weeks of age, body weight ~25 ± 2 g) were purchased from Jinan Pengyue Animal Breeding Co., LTD. (Jinan, China). The animal experiments were performed in accordance with international regulations and were approved by the Shandong Laboratory of Yantai Drug Discovery Institutional Animal Care and Use Committee (IACUC number: 2023-05-XX-472, approval date: 17 May 2023). Mice were housed in standard polypropylene cages (3 mice per cage) under controlled environmental conditions: temperature 22–24 °C, relative humidity 40–60%, and a 12 h light/12 h dark cycle. Before the experiment, mice were acclimatized for 7 days with free access to standard chow and sterile water.

### 2.3. Animal Experiments

In total, 36 male *C57BL/6J* mice, 8 weeks of age and weighing approximately 25 ± 2 g, were placed in isolated animal cages under standard laboratory conditions (ambient temperature of 22–24 °C, relative humidity of 40–60%, and a light-dark cycle of 12-12 h) and were permitted to eat and drink freely during the experiments. *C57BL/6J* male mice were randomly divided into two groups:

(1) High-fat diet + vehicle (HFD + vehicle): The mice received a daily intragastric administration of PBS with the high-fat diet for a period of 55 days. Mouse body weight and food intake per cage were measured every 5 days.

(2) In the second group, designated the “HFD + DB (10 mg/kg/d)” group, the mice received a daily intragastric administration of 10 mg/kg DB with the high-fat diet for a period of 55 days. Mouse body weight and food intake per cage were measured every 5 days.

At 24 h after the final dose, the mice were anesthetized with pentobarbital, then individual blood and muscle samples were collected from each mouse. All samples were stored at −80 °C until assayed. The DIO model and preventive treatment protocol were adapted from Surwit et al. [24].

The nutritional composition of the HFD provided by Shanghai Bioleaf Biotech Co., Ltd. (Shanghai, China) is shown in Table 1.

### 2.4. Cell Culture

Mouse 3T3-L1 preadipocytes (purchased from the Cell Bank of the Chinese Academy of Sciences, Shanghai, China) were cultured in DMEM containing 10% FBS and 1% penicillin/streptomycin (P/S) at 37 °C in a 5% CO_2_ incubator, and fully fused cells were cultured in differentiation induction medium for 48 h. The change in induction medium was recorded on day 0, consisting of 0.172 µM insulin (Tsbiochen T8221), 0.25 µM dexamethasone (Tsbiochen T6465) and 0.5 mM 3-isobutyl-1-methylxanthine (Tsbiochen T1713), as was the change in maintenance medium on day 2 (consisting of 10% FBS, 1% penicillin/streptomycin and 0.172 µM insulin). All compounds were added to the induction and maintenance medium on day 0, and the maintenance medium was changed every other day thereafter [25].

In the present study, 3T3-L1 cells were exposed to various concentrations of DB during the differentiation process. The expression of Ucp1 was evaluated at the conclusion of the differentiation process.

### 2.5. White Adipocyte Isolation and Differentiation

Primary white adipocytes were isolated and induced according to the protocol described [26]. Mice were anesthetized with pentobarbital, and inguinal adipose tissue was subsequently harvested. The inguinal white adipose tissues (iWATs) were extracted from five 10-week-old *C57BL/6J* male mice. These were then digested in HEPES-based buffer, containing a specific collagenase enzyme, at 37 °C. Following this step, the samples were then subjected to a centrifugation process at 1500 rpm for 15 min at 4 °C. The resulting pellet was resuspended in DMEM containing 10% FBS and 4% penicillin/streptomycin (P/S) and filtered through a 70 μm nylon mesh, and the number of cells was counted using a cell counter plate. The filtered solution was then inoculated into 6-well plates at a density of 2 × 10^6^ cells/well at 37 °C with 5% CO_2_. Following a 24 h incubation period, the cells were rinsed with PBS and then incubated in a growth medium (DMEM containing 10% FBS and 1% penicillin/streptomycin (P/S)) at 37 °C with 5% CO_2_. The medium was changed every other day until the cells reached confluence.

Following confluence, the preadipocytes were induced by changing the medium to an induction medium (DMEM with 10% FBS, 1% penicillin-streptomycin, 0.172 µM insulin, 0.25 µM dexamethasone, and 0.5 mM 3-isobutyl-1-methylxanthine) over a period of 48 h (day 0 to day 2). Thereafter, from day 2 to day 12, cells were maintained in a maintenance medium (DMEM with 10% FBS, 1% penicillin-streptomycin, 0.172 µM insulin), which was renewed every other day. Cells were treated chronically with different concentrations of DB starting on day 0. Media and treatments were changed every other day. Cells were analyzed on day 12. It is noteworthy that all experiments involving preadipocytes utilized cells on day 0.

### 2.6. Oil Red O and Hematoxylin Staining

Following the differentiation of adipocytes, the cell culture solution was aspirated gradually and the samples washed with PBS once. Thereafter, 4% paraformaldehyde fixative was added and the samples were left to fix for 10 min. The fixative was then rinsed twice with PBS. The cells were then covered with an appropriate amount of 60% isopropanol washing solution for 20 s, after which the washing solution was aspirated. Thereafter, an appropriate amount of Oil Red O staining solution was added and the cells were stained for 10–20 min. The Oil Red O staining solution was then removed, and the cells were washed with 60% isopropanol for 30 s. Finally, the cells were washed with PBS for a further 20 s. The nuclei were then restained with hematoxylin staining solution for a duration of 30 s. The hematoxylin staining solution was then removed and the cells were washed with PBS. The cells were then covered with PBS and observed and photographed by microscope. The above experimental protocol refers to the references [27,28].

### 2.7. Total Protein Extraction and Western Blotting

Total protein extraction and Western blotting were performed as previously described [29]. Cells were homogenized in RIPA buffer containing 1% protease inhibitor cocktail and 1% phosphatase inhibitor cocktail (Thermo Fisher Scientific) on ice for 30 min. After centrifugation at 10,000× *g* for 30 min at 4 °C, the supernatant was collected as total protein extract. Protein concentration was determined using the Bradford reagent (Thermo Fisher Scientific), with bovine serum albumin (BSA) as the standard. A total of 20 µg of protein per sample was separated by 10% SDS-PAGE electrophoresis, then transferred onto polyvinylidene fluoride (PVDF) membranes (Millipore, Burlington, MA, USA).

The membranes were blocked with 5% non-fat milk in TBST (Tris-buffered saline with 0.1% Tween-20) for 1 h at room temperature. Primary antibodies (anti-Ucp1, anti-*PRDM16*, anti-GAPDH, all 1:1000 dilution) were incubated with the membranes overnight at 4 °C. Membranes were washed three times with TBST (10 min each), then secondary antibodies (horseradish peroxidase-conjugated goat anti-rabbit IgG, 1:5000 dilution, Abcam) were incubated with the membranes for 1 h at room temperature. Membranes were washed three times with TBST (10 min each). The protein bands were detected using an Amer Sham Imager 600 (GE, Boston, MA, USA), followed by a quantitative analysis with ImageJ 1.53t software (National Institutes of Health, MD, USA). GAPDH was employed as an internal control.

### 2.8. Real-Time Fluorescence Quantitative PCR Analysis

Total RNA was isolated from cells and tissues using TRIzol Reagent (Vazyme R401-01). Following this, the samples were treated with RNase-free DNase and reverse-transcribed to cDNA using 5× All-in-One RT Master Mix (Vazyme R333-01) in accordance with the manufacturer’s protocols. The quantification of cDNA levels was performed using 2× ChamQ Blue Universal SYBR qPCR Master Mix (Vazyme Q312-02). RT-qPCR (Roche Lightcycler* 96, Mannheim, Germany) was then utilized to quantify the gene transcription levels. The quantitative polymerase chain reaction (qPCR) assay was performed to standardize the transcript levels of all genes to the glyceraldehyde-3-phosphate dehydrogenase (GAPDH) level. The primer sequences utilized in this study are delineated in Table 2.

### 2.9. Blood Parameter Analysis

Mouse blood was collected via enucleation. For serum collection, the blood was left to clot at room temperature for 1 h, followed by centrifugation at 13,000× *g* for 15 min at 4 °C. Serum triglyceride (TG), total cholesterol, and low-density lipoprotein (LDL) cholesterol levels were measured using an automatic biochemical analyzer (JCA-BM6010/C).

### 2.10. Detection of GLP-1 and CCK in Mouse Plasma

The plasma samples were taken out from storage at −80 °C, thawed at room temperature, then placed on a vortex oscillator to mix for 10 s (avoiding violent oscillation which may cause protein denaturation). The experimental operations were performed according to the instructions of the mouse GLP-1/CCK enzyme-linked immunosorbent assay (ELISA) kit. Finally, the absorbance (OD value) was measured at a wavelength of 450 nm using a microplate reader, and the content of mouse GLP-1/CCK in the samples was calculated through the standard curve. 

### 2.11. Data Analysis

Data are presented as mean ± standard error of the mean (S.E.M). All analyses were performed using GraphPad Prism 6 software (GraphPad Software, La Jolla, CA, USA; www.graphpad.com).

(1) Comparison of body weight change and gene expression levels between two groups (HFD + vehicle vs. HFD + DB) was conducted using two-tailed Student’s *t*-test.

(2) Comparison of multiple groups (e.g., different DB concentrations and different time points) was conducted using one-way analysis of variance (one-way ANOVA) followed by Tukey’s multiple comparisons test.

(3) Analysis of body weight change over time with group effects was conducted using two-way ANOVA followed by Sidak’s multiple comparisons test.

All comparisons are reported with 95% confidence intervals. *p* < 0.05 was considered statistically significant, with *p* < 0.05 marked as *, *p* < 0.01 as **, and *p* < 0.001 as ***.

## 3. Results

### 3.1. DB Reduces HFD-Induced Obesity in Mice

**Figure 1:**
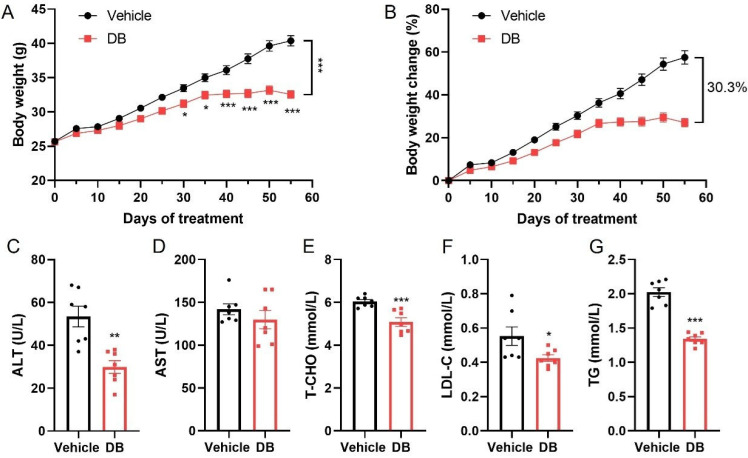
Effects of DB on body weight in high-fat diet (HFD) mice. Eight-week-old male *C57BL/6J* mice were fed a high-fat diet for 55 days and received a daily intragastric administration of either PBS (vehicle) or 10 mg/kg DB. During the DB treatment, body weight was measured every 5 days. At 24 h after the final dose, the mice were euthanized, and plasma was collected. The effects on body weight (**A**) and the rate of body weight change (**B**) were monitored. Data are presented as mean ± S.E.M. (n = 18) and were analyzed by two-way ANOVA with multiple comparisons test. * *p* < 0.05, *** *p* < 0.001 vs. vehicle. Plasma alanine aminotransferase (ALT), aspartate aminotransferase (AST), total cholesterol (T-CHO), low-density lipoprotein cholesterol (LDL-C) and triglycerides (TGs) were measured (**C**–**G**). Data are presented as mean ± S.E.M. (n = 7) and were analyzed by two-tailed Student’s *t*-tests. * *p* < 0.05, ** *p* < 0.01, *** *p* < 0.001, vs. vehicle.

To investigate the effects of DB on the body weight of HFD-induced obese mice, we selected 8-week-old male *C57BL/6J* mice with similar baseline body weights. These mice were fed a high-fat chow and simultaneously administered daily intragastric gavage of 10 mg/kg DB. Body weight measurements were conducted every five days. As illustrated in Figure 1A,B, DB treatment effectively prevented further body weight gain in HFD-fed mice compared to the PBS-treated control group. From day 30 onward, the body weight gain of DB-fed mice was significantly lower than that of the control animals. At day 55, the body weight change of DB-fed mice was 30.3% lower than that of the control group. Furthermore, as shown in Figure 1C–G, serum concentrations of ALT, total cholesterol (T-CHO), LDL-C and TG were significantly lower in DB-treated mice compared to the PBS group, indicating that long-term DB administration could reduce body weight gain, reduce lipid levels and enhance liver function in HFD-fed mice.

### 3.2. DB Does Not Affect Food Intake and Gut Hormones

**Figure 2:**
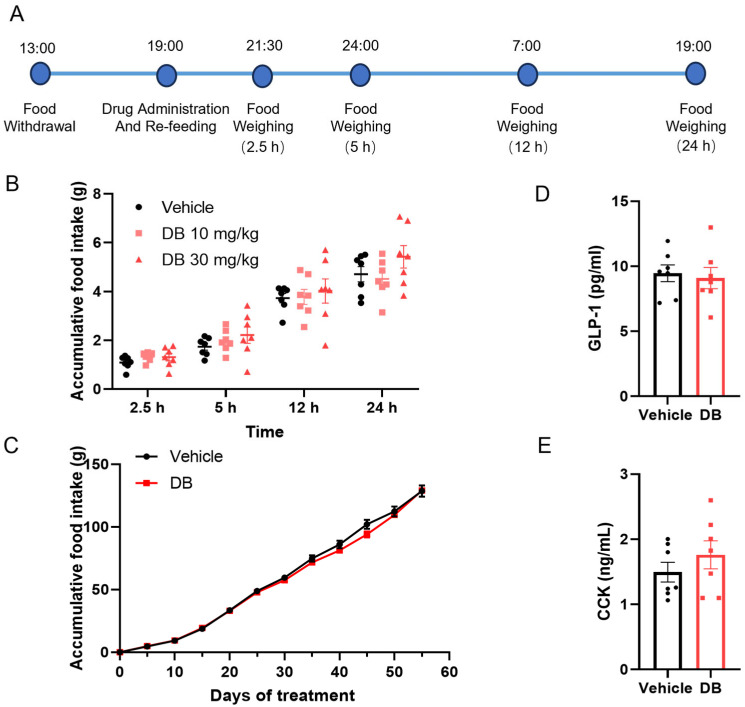
DB does not affect food intake and gut hormones. (**A**) Schematic diagram of the acute feeding experiment in mice. (**B**) Effects of DB on acute food intake. On the day before the experiment, mice were housed individually (one per cage). At 13:00, the food was removed, and the mice were fasted for 6 h. At 19:00, the mice were administered PBS (vehicle) or DB (10 mg/kg and 30 mg/kg) by gavage, and the food was simultaneously returned. Food intake was measured by weighing the remaining food at 2.5, 5, 12, and 24 h after DB administration to calculate cumulative food consumption. Data are presented as mean ± S.E.M. (n = 7). (**C**) Effects of DB on chronic food intake. Eight-week-old male *C57BL/6J* mice were fed a high-fat diet for 55 days and received a daily intragastric administration of either PBS (vehicle) or 10 mg/kg DB. Food intake was measured during the treatment. Data are presented as mean ± S.E.M. (n = 6). (**D**,**E**) Effects of DB on gut hormones. *C57BL/6J* mice were fed a high-fat diet for 55 days and received a daily intragastric administration of either PBS (vehicle) or 10 mg/kg DB. At 24 h after the final dose, the mice were euthanized, and plasma was collected to measure the levels of active GLP-1 (**D**) and CCK (**E**). Data are presented as mean ± S.E.M. (n = 7).

Considering that DB is a strong bittering agent and commonly added into household products to prevent ingestion of harmful substances through taste aversion, we wondered whether it prevents weight gain by reducing food intake. As shown in Figure 2B,C, neither acute nor chronic intragastric administration of DB significantly affected food intake. To investigate potential mechanisms, we measured intestinal hormone levels after 55 days of treatment and found no significant differences in GLP-1 or cholecystokinin (CCK) concentrations between DB-treated and control groups (Figure 2D,E). These data collectively indicate that DB-mediated weight control in HFD-induced obesity does not involve alterations in food consumption or major satiety hormone signaling. This highlights alternative metabolic pathways as likely targets for DB’s beneficial effects on obesity.

### 3.3. DB Enhances Ucp1 Expression in Muscle and 3T3-L1 Cells

**Figure 3:**
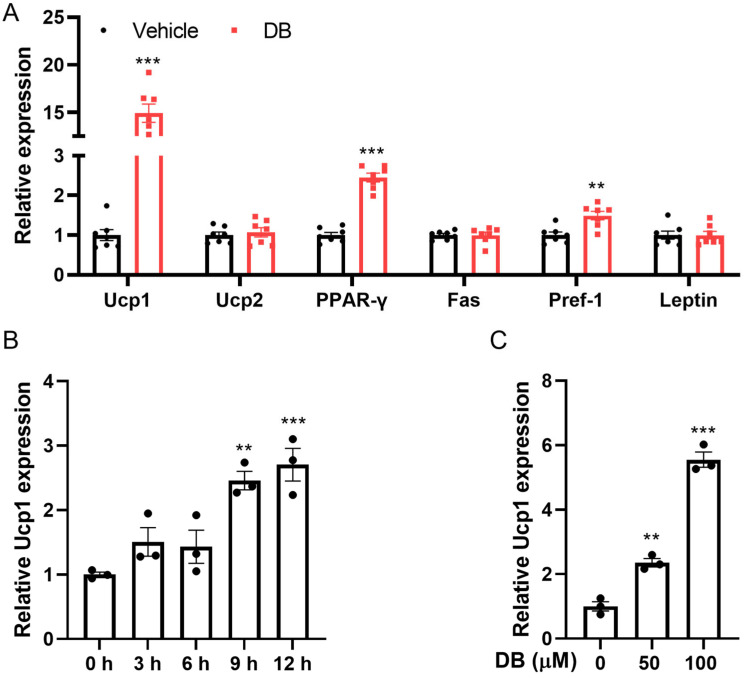
DB increases the expression of *Ucp1* in mice and 3T3-L1 cells. (**A**) Mice were fed a high-fat diet for 55 days and received daily intragastric administration of either PBS (vehicle) or 10 mg/kg DB. The relative mRNA expression levels of *Ucp1*, *Ucp2*, *PPAR-γ*, *Fas*, *Pref-1*, and *Leptin* were measured in the skeletal muscle after the final dose. Data are presented as mean ± S.E.M. (n = 7) and were analyzed by two-way ANOVA with multiple comparisons test. ** *p* < 0.01, *** *p* < 0.001 vs. vehicle. (**B**) The expression of *Ucp1* in 3T3-L1 cells was measured after treatment with 100 μM DB at various time points. (**C**) Following a 12-day treatment of 3T3-L1 cells with DB at concentrations of 50 and 100 μM, *Ucp1* expression was analyzed using RT-qPCR. Data are presented as mean ± S.E.M. (n = 3) and were analyzed by one-way ANOVA with multiple comparisons test. ** *p* < 0.01, *** *p* < 0.001 vs. control.

Given that skeletal muscle constitutes approximately 40% of total body mass in humans and accounts for about 30% of whole-body basal metabolic rate (BMR) under resting conditions, it is recognized as one of the principal metabolic organs [33]. To investigate potential effects on metabolism following prolonged treatment, we extracted total RNA from the skeletal muscles of mice subjected to DB administration for 55 consecutive days, and measured the expression of metabolism-related genes, including *Ucp1*, *Ucp2*, *PPAR-γ*, *Fas*, *Pref-1*, and *Leptin*. We observed a significant upregulation of the key marker of brown fat, gene *Ucp1*, and the key marker of adipogenesis, *PPAR-γ*, in the DB group relative to the PBS-treated control group (Figure 3A).

Since *Ucp1* plays a critical role in heat generation in adipocytes, we wondered whether DB promotes the expression of *Ucp1* in 3T3-L1, a cell commonly used to study adipogenesis. As illustrated in Figure 3B, treatment with 100 μM DB significantly elevated *Ucp1* mRNA levels compared with the control, with the most pronounced increase observed at the 12 h time point. Furthermore, during the differentiation of 3T3-L1 cells, DB was administered at varying concentrations. The results demonstrated a clear concentration-dependent upregulation of *Ucp1* expression (Figure 3C). Collectively, these data indicate that DB enhances *Ucp1* expression not only in skeletal muscle but also in 3T3-L1 adipocytes.

### 3.4. DB Promotes Browning of 3T3-L1 Cells and Primary White Adipocytes

**Figure 4:**
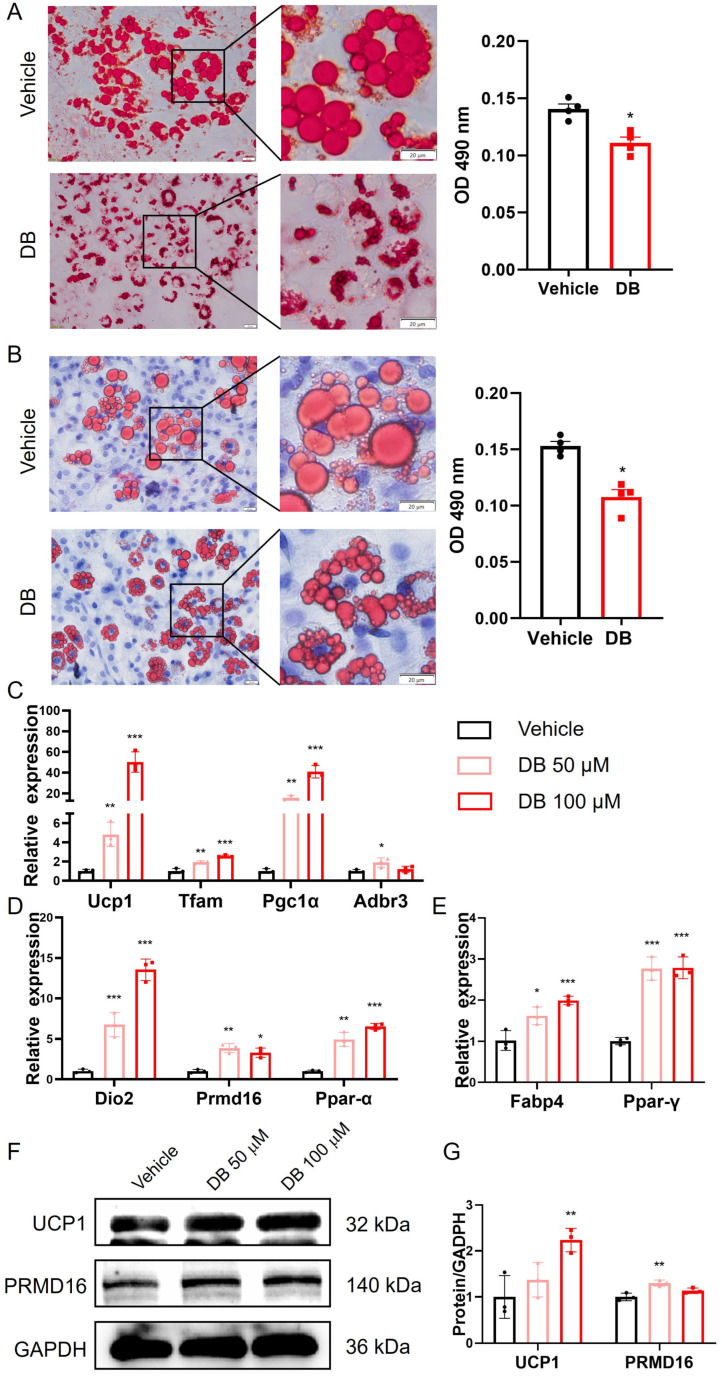
DB enhances browning of 3T3-L1 cells and white adipocytes. (**A**,**B**) The effect of DB on adipocyte differentiation. 3T3-L1 preadipocytes and primary white adipocytes were differentiated for 0–12 days in induction medium containing either vehicle or 100 μM DB. On day 12, cells were fixed with 4% paraformaldehyde and stained with Oil Red O to visualize neutral lipid accumulation. Representative photomicrographs of Group 3T3-L1 cells (**A**) and primary white adipocytes (**B**) are shown, Scale bars, 20 μm. Quantitative analysis of Oil Red O staining was performed to assess lipid content on the right. Data are presented as mean ± S.E.M. and were analyzed by two-tailed Student’s *t*-tests. * *p* < 0.05, vs. vehicle. n = 4 biologically independent samples. (**C**–**E**) Quantitative analysis of gene expression in mature primary white adipocytes. Cells were treated with or without DB and the relative mRNA levels of the thermogenic markers *Ucp1*, *TFAM*, *Pgc1α*, and *Adrb3* (**C**), the adipose browning markers *Dio2*, PRMD16, and *PPAR-α* (**D**), and the adipogenic markers *Fabp4* and *PPAR-γ* (**E**) were determined. Data are presented as mean ± S.E.M. (n = 3) and were analyzed by two-way ANOVA with multiple comparisons test. * *p* < 0.05, ** *p* < 0.01, *** *p* < 0.001 vs. vehicle. (**F**,**G**) Quantitative analysis of protein levels in mature primary white adipocytes. Cells were treated with or without DB and the protein levels of the brown adipocyte markers *Ucp1* and PRMD16 were measured by Western blot. Representative immunoblot (**F**) and quantification (**G**). Data are presented as mean ± S.E.M. (n = 3) and were analyzed by two-way ANOVA with multiple comparisons test. ** *p* < 0.01, vs. vehicle.

To assess the effect of DB on adipocyte differentiation, 3T3-L1 cells and primary white adipocytes undergoing differentiation were treated with DB. As shown in Figure 4A,B, DB-treated cells contained numerous small, multilocular lipid droplets, as visualized by Oil Red O staining. The quantitative analysis of lipid content at OD 490 revealed a marked reduction in lipid accumulation compared with the controls. These morphological features are characteristic of brown-like adipocytes.

We next examined mRNA expression levels in mature primary white adipocytes. DB treatment significantly upregulated the thermogenic markers *Ucp1*, *TFAM*, *Pgc1α*, and *Adrb3* (Figure 4C); the adipose browning markers *Dio2*, PRMD16, and *PPAR-α* (Figure 4D); and the adipogenic markers *Fabp4* and *PPAR-γ* (Figure 4E). Consistent with these findings, Western blot analysis demonstrated a significant increase in the protein levels of the brown adipocyte markers *Ucp1* and Prmd16 following DB treatment (Figure 4F,G). Collectively, these results indicate that DB promotes both the thermogenic activation and browning of white adipocytes.

## 4. Discussion

The bitter compound DB has demonstrated efficacy in inhibiting weight gain in mice fed a high-fat diet [16]. Our study reaffirms the weight-reducing effects of DB in this model, along with its ability to lower blood lipid levels and enhance liver function. Unlike many existing diet medications that function by curbing appetite and decreasing food consumption [34,35], DB operates through a distinct mechanism.

The intestine, as the primary organ for digestion and absorption, plays a pivotal role in modulating energy intake by secreting specific peptides [13]. Among these, glucagon-like peptide-1 (GLP-1) and cholecystokinin (CCK) are key regulators [36]. GLP-1, originating from intestinal L cells, can reduce appetite and food consumption, consequently promoting weight loss [37]. Research has demonstrated that administering DB intragastrically to mice can enhance GLP-1 release [38,39], suppressing appetite and reducing food intake [15]. On the other hand, CCK, produced by intestinal I cells, binds to intestinal CCK1 receptors, slowing gastric emptying. Moreover, CCK communicates with the hypothalamus through the vagus nerve, heightening satiety signals and decreasing food consumption [36]. In STC-1 cells, DB has been shown to elevate intracellular Ca^2+^ levels in a dose-dependent manner, stimulating CCK secretion [40,41]. Our investigation found no significant disparity in food intake between mice treated with DB and control subjects following prolonged DB administration. We evaluated the expression of gut hormones, particularly GLP-1 and CCK, after extended exposure to DB. While GLP-1 levels remained consistent across groups, there was a slight non-significant increase in CCK levels in DB-treated mice. Bert et al. similarly noted minimal impacts of DB and quinine on gut hormones in mice [16]. These observations may stem from regulatory variances between whole organisms and in vitro cell models. Consequently, it is plausible that DB’s weight loss effects are not mediated through the regulation of food intake.

Adipose tissue metabolism and differentiation are crucial in obesity. Brown and beige adipose tissues are key sites for thermogenesis, enhancing energy metabolism and reducing lipid accumulation, thus mitigating cardiovascular disease risk primarily via *Ucp1* [42]. Studies indicate that bitter compounds influence adipocytes. In 3T3-L1 cultures, caffeine reduces lipid accumulation by inhibiting insulin-stimulated glucose uptake and promoting lipolysis. Nobiletin increases the expression of brown adipocyte markers (*Pgc1α* and *Ucp1*) and fatty acid oxidation genes, along with PKA and AMPK phosphorylation [43]. In *C57BL/6J* mice, the daily oral administration of epicatechin increases brown fat-specific marker proteins in perivisceral subcutaneous adipose tissue [44]. Dietary luteolin has been shown to upregulate thermogenic genes in both brown and subcutaneous adipose tissues, while also enhancing PGC-1α expression and AMPK phosphorylation [45]. In human adipose-derived stem cells, silibinin elevates *Ucp1* thermogenic marker gene expression and reduces lipid content [46]. DB, noted for its extreme bitterness, significantly upregulates *Ucp1* and *PPAR-α* expression in mouse muscle tissues. In 3T3-L1 cells, DB increased the expression of the *Ucp1* gene in a concentration- and time-dependent manner. Avau et al. reported that DB reduced lipid accumulation in 3T3-F442A adipocytes by regulating lipid metabolism [16]. By adding DB during the differentiation induction of 3T3-L1 and mouse primary preadipocytes, we found that DB treatment could lead to a decrease in the size of lipid droplets in adipocytes. Meanwhile, the expression of adipogenic marker genes *Fabp4* and *PPAR-γ*; thermogenic marker genes *Ucp1*, *TFAM*, *Pgc1α*, and *Adrb3*; and browning marker genes *Dio2*, *PRDM16*, and *PPAR-α* was significantly increased. *PRDM16* and *Pgc1α* cooperate with the general adipogenic transcription factor *PPAR-γ* to promote the differentiation of beige adipocytes [32]. Laia et al. found that an EPAC1 agonist could increase lipid droplets in preadipocytes, induce the expression of thermogenic genes *Ucp1* and *Pgc1α*, increase the level of mitochondrial complex OXPHOS, and promote the generation of brown adipocytes [26]. DB treatment could increase the expression of brown adipocyte marker proteins *Ucp1* and *PRDM16*. These results indicate that DB can promote the differentiation of adipocytes into beige adipocytes.

Bitter taste receptors, as the sites of action for bitter compounds, are widely distributed in various tissues and organs and are associated with multiple diseases such as obesity and diabetes [47]. A study by Ning et al. showed that adding quinine to the differentiation medium of primary mouse preadipocytes enhanced lipid accumulation in differentiated adipocytes. The expression of Tas2r106 in adipocytes increased, and knocking down Tas2r106 inhibited quinine-mediated adipogenesis [48]. The overexpression of Tas2r108 or Tas2r126 resulted in decreased lipid accumulation, the downregulation of the adipocyte marker genes *PPAR-γ* and Cebpa, and the inhibition of mature adipocyte differentiation in 3T3-L1 cells [49]. Given the intricate genetic variability of bitter taste receptors and the ambiguity surrounding their ligands, additional research is warranted to elucidate whether DB mediates its effects via bitter taste receptors and to identify the specific receptors implicated.

## 5. Conclusions

In summary, this study elucidates a novel mechanism underlying DB-induced weight reduction. Unlike previous studies that linked DB to appetite suppression via GLP-1 and CCK signaling, our results demonstrate that DB does not modulate food intake through these satiety hormones. Instead, our data reveal that DB activates the browning program in adipocytes, characterized by enhanced *Ucp1* expression, thereby increasing whole-body energy expenditure. This unexpected finding not only challenges the prevailing view of DB’s primary mode of action but also highlights adipose tissue thermogenesis as a promising alternative therapeutic target for obesity management. Future investigations should explore the upstream signaling pathways linking DB to *Ucp1* transcription and validate these effects in clinical populations.

## Figures and Tables

**Table 1:** The nutritional composition of the high-fat diet (HFD) used in the study.

Nutrient Category	Specific Nutritional Indicators	Content/Specification (Catalogue No.: D12492)
Energy Supply	Fat Energy Ratio	60 kcal%
	Fructose Energy Ratio	No fructose added
	Basic Energy	5.21 kcal/g (dry weight)
Core Nutrients	Cholesterol	0.15%
	Fat Source	Palm oil and shortening (ratio 1:1)
Basic Nutrients	Crude Protein	185 g/kg
	Crude Fiber	52 g/kg
	Crude Ash	78 g/kg
	Moisture	≤95 g/kg
Vitamins and Minerals	Composite Vitamins Composite Minerals	Vitamin A (15000 IU/kg), Vitamin D3 (1500 IU/kg), Vitamin E (100 mg/kg), Vitamin B (adequate) Calcium (12 g/kg), Phosphorus (8 g/kg), Iron (150 mg/kg), Zinc (60 mg/kg)

**Table 2:** List of primers used for real-time quantitative PCR.

Gene	Primer Sequence	Reference
*Ucp1-F*	TAAGCCGGCTGAGATCTTGT	[26]
*Ucp1-R*	GGCCTCTACGACTCAGTCCA	
*TFAM-F*	CCTTCGATTTTCCACAGAACA	
*TFAM-R*	CCTTCGATTTTCCACAGAACA	
*PPAR* *-γ-* *F*	ACTGCAGCCCCCTATAGT	
*PPAR-γ-R*	GGATCAGTTGGGTCAGTGGG	
*Fabp4-F*	TGAAAGAAGTGGGAGTGGGCTTTGC	
*Fabp4-R*	CACCACCAGCTTGTCACCATCTCGT	
*Adrb3-F*	CCTTCAACCCGGTCATCTAC	
*Adrb3-R*	GAAGATGGGGATCAAGCAAGC	
*P* *PAR-* *α-F*	GCGTACGGCAATGGCTTTAT	[30]
*P* *PAR-* *α-R*	GACAAATGCTCTTTGCTTTATTGC	
*Ucp2-F*	TTGCCTCCCCCGTTGAT	
*Ucp2-R*	TACTGGCCCAAGGCAGAG	
*Fas* *-F*	GGACATGGTCACAGACGATGAC	
*F* *as* *-R*	GTCGAACTTGGACAGATCCTTCA	
*Pref-1-F*	CTGTTTACAAACAATATCCGA	
*Pref-1-R*	CGACCCTCTGTGAAGTTGGTG	
*Leptin-F*	TGACACCAAAACCCTCATCA	[31]
*Leptin-R*	AGCCCAGGAATGAAGTCCA	
*Dio2-F*	CAGTGTGGTGCACGTCTCCAATC	[32]
*Dio2-R*	TGAACCAAAGTTGACCACCAG	
*P* *RDM* *16-F*	CAGCACGGTGAAGCCATTC	
*P* *RDM* *16-R*	GCGTGCATCCGCTTGTG	
*Pgc1α-F*	ACCCACAGGATCAGAACAAAC	
*Pgc1α-R*	TGTGTCGAGAAAAGGACCTTGA	
*GAPDH-F*	TGGTCTCCTCTGACTTCAAC	
*GAPDH-R*	GTGAGGGTCTCTCTCTTCCT	

## Data Availability

The original contributions presented in this study are included in the article. Further inquiries can be directed to the corresponding authors.
